# The Role of Cardiovascular Magnetic Resonance in Pediatric Congenital Heart Disease

**DOI:** 10.1186/1532-429X-13-51

**Published:** 2011-09-21

**Authors:** Hopewell N Ntsinjana, Marina L Hughes, Andrew M Taylor

**Affiliations:** 1 Centre for Cardiovascular MR, UCL Institute of Cardiovascular Sciences, Great Ormond Street Hospital for Children, London, UK

## Abstract

Cardiovascular magnetic resonance (CMR) has expanded its role in the diagnosis and management of congenital heart disease (CHD) and acquired heart disease in pediatric patients. Ongoing technological advancements in both data acquisition and data presentation have enabled CMR to be integrated into clinical practice with increasing understanding of the advantages and limitations of the technique by pediatric cardiologists and congenital heart surgeons. Importantly, the combination of exquisite 3D anatomy with physiological data enables CMR to provide a unique perspective for the management of many patients with CHD. Imaging small children with CHD is challenging, and in this article we will review the technical adjustments, imaging protocols and application of CMR in the pediatric population.

## 1. Introduction

Congenital heart disease (CHD) has an incidence of 6-8 per 1000 at birth [1,2]. The survival of CHD patients has also increased because of improvements in early diagnosis (including fetal echocardiography) and treatment, which have led to more patients surviving into adulthood [1-3]. Furthermore, there is an increasing number of children with acquired heart disease, in particular related to anthracycline cardiotoxicity, following treatment of oncological disease in early childhood.

Imaging is fundamental to the diagnosis of CHD and is required at all stages of patient care. From the fetal stage onwards, imaging outlines anatomy and physiology, helps to refine management, evaluates the consequences of interventions and helps guide prognosis. However, no single available imaging modality fulfils these roles for all patients and diseases. ***Therefore, assessment for CHD must involve a variety of modalities that can be used in a complementary fashion, and that together are sensitive, accurate, reproducible, and cost effective, whilst minimizing harm***.

Echocardiography remains the first-line imaging investigation for pediatric patients, as it is portable, non-invasive and provides immediate, high-resolution anatomical and physiological information [4,5]. For co-operative patients with good acoustic windows, echocardiography alone can define diagnosis and guide management and prognosis. However, echocardiography fails when acoustic windows are poor, particularly for the assessment of extra-cardiac vascular structures.

Where cardiac catheterization was traditionally used to provide hemodynamic information and visualize extracardiac great vessels, [6] cardiovascular MR (CMR) is progressively fulfilling this role [7]. The burgeoning availability of MR scanners and physicians' rapid uptake of CMR is escalating the prominence of this modality in the management of pediatric congenital heart disease.

CMR provides a powerful tool, giving anatomical and physiological information that echocardiography and catheterization alone cannot provide [8,9]. Extra-cardiac anatomy, including the great arteries, systemic and pulmonary veins, can be delineated with high spatial resolution. Vascular and valvular flow can be assessed, [10] shunts can be quantified, [11] and myocardial function can be measured accurately with high reproducibility, regardless of ventricular morphology [12]. Finally, CMR surpasses both catheterization and echocardiography in providing high-resolution, isotropic, three-dimensional datasets [13]. This allows for reconstruction of data in any imaging plane, giving complete visualization of complex cardiac anomalies, without the use of ionizing radiation [14]. ***In the pediatric population, CMR could be justified for any patient in whom clinical or echocardiographic data is insufficient for monitoring, decision-making or surgical planning***.

Despite its widespread use CMR still has some technical limitations that have to be overcome in order to perform successful pediatric CMR. These technical difficulties involve the high spatial resolution required for imaging small anatomical structures, and the patients' inability to consistently follow breath-holding commands, due to young age or developmental delay. This review will aim to provide guidance on the indications for CMR in pediatric CHD, provide potential protocols and describe imaging techniques for the main conditions referred for CMR.

## 2. Indications

The decision to perform CMR depends on the information required, the local facilities and resources available for scanning, the clinical state of the patient, and the risks to the patient of carrying out the examination. Without the use of sedation or contrast, a comprehensive CMR examination in a willing patient carries minimal risk. However, the need for sedation, general anesthesia or gadolinium contrast changes the balance of risk in some patients. Furthermore, CMR is a resource-high investigation. In addition to the costs of purchasing, running and maintaining the MR scanner, significant expertise and training is required for all staff involved in acquiring and interpreting the images.

The technical and diagnostic complexity of pediatric CMR is significant. The patients' body size is small and heart rates are rapid. Imaging these patients requires a radiographer trained to expedite image planning and optimize pulse sequences in this context, and a CMR physician with expertise in the anatomical and physiological changes of CHD. In addition, because general anesthesia is often necessary for the youngest children, an anesthetic team is required, and this team must be trained to care for cardiac patients with hemodynamic compromise.

### 2.1. CMR with general anesthesia

Because of the potential increased risks involved in pediatric patients with congenital heart disease, in our institution, the decision for a child to undergo CMR under general anesthesia is made in the setting of a multidisciplinary clinical planning meeting. The decision-making involves careful analysis of potential risks and benefits. Our unit policy is that a senior cardiac anesthesiologist always carries out the anesthetic procedure. Prior to each case there is detailed discussion between the anesthetic and cardiac imaging teams, regarding the specific hemodynamic and imaging issues pertaining to the case. With these considerations, our unit and others, have a very good safety profile for imaging these complex patients [15,16].

Generally, children less than seven years of age will have CMR performed under a general anesthetic. This practice varies in different centers, depending on local anesthetic and sedation policy. Some institutions use various degrees of sedation, with or without the need for an anesthetist to monitor the patient. General anesthesia ensures prolonged cooperation and enables reliable breath holding.

**Figure 1:**
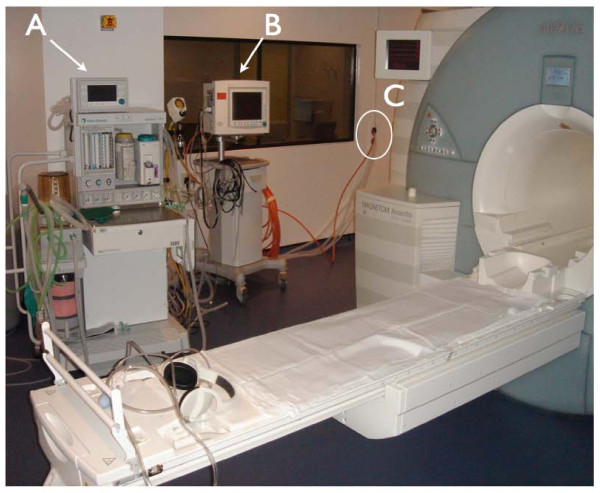
**CMR set-up for paediatric general anaesthetic cases**. View of the MR scanner room showing the anesthetic machine **(A) **and monitoring equipment **(B)**. Ventilation tubing and leads from both pieces of equipment pass through a small opening in the wall **(C) **into the control room, so that the anesthetist can control breath-holding and monitor the patient from within the control room.

Potential indications for children undergoing CMR under general anesthesia are outlined in Additional file 1, Table S1. The set-up of a typical CMR control room containing anesthetic equipment is shown in Figure 1.

Other procedures can be carried out while the patient is under anesthetic. For example, in those patients with a functionally uni-ventricular heart and a cavo-pulmonary shunt, the jugular venous pressure can be measured via needle transducer, prior to surgical completion of the total cavo-pulmonary circulation. This gives an estimation of pulmonary artery pressure at the same time that image data gives pulmonary artery morphology, flow volume, ventricular and valvular function. Diagnostic catheterization can be avoided in many patients who have traditionally required catheter angiography [17,18].

### 2.2 CMR without anesthesia

The older pediatric patient groups for whom CMR is indicated are listed in Additional file 2, Table S2. For many of these patients CMR is often a single, focused study prior to intervention. For others the benefit of CMR lies in serial imaging leading up to, or following intervention. While avoiding ionizing radiation, CMR can give accurate and reproducible quantification aortic arch dimensions [19,20], ventricular volumes, and valvular function [21]. This guides the management team with regards to the appropriate timing for, [22] or the effect of any intervention [23,24].

### 2.3 Prior to transfer to adult services

An important indication for CMR in our pediatric centre is the stage of transfer of the patient to an adult institution for ongoing care. Prior to transfer, CMR gives a comprehensive summary of the anatomical and physiological status of the patient, for all types of post-surgical situations.

### 2.4 Decision making

When there are local facilities and expertise in all the modalities: CMR, cardiovascular CT and cardiac catheterization, the imaging strategy for complex patients can be discussed in a forum comprising cardiologists, cardiac imaging specialists, interventionists and surgeons.

For many patients the imaging choice is obvious. For example, for a cooperative 10 year old with clinical signs of recurrent aortic coarctation, following repair in infancy, CMR would yield high-resolution images of the aortic arch morphology and give the flow profile through the arch. At the same time, the CMR would portray and quantify aortic valve function and left ventricular myocardial structure, mass and systolic function. This data could be acquired within 40 minutes of scanning time, with no need for sedation, anesthetic or irradiation.

One could argue that for this patient, cardiac catheterization could provide data on the arch morphology and give an opportunity for arch intervention. However, the best mode and timing of intervention is not always clear for many patients. Imaging, with a subsequent temporal pause or "discussion window" for consideration of all management options would most frequently yield the optimal outcome.

At the other end of the risk-benefit spectrum for comprehensive imaging is an infant with hypoplastic left heart syndrome (HLHS), clinically deteriorating soon after the first stage of surgical palliation. With poor acoustic windows, urgent further imaging of the branch pulmonary arteries and aortic arch is necessary. In this context, general anesthetic may carry a high risk, and CT imaging of the chest would usually be performed, using a non-sedated "feed and wrap" technique. The CT images would then be used to refine the decision-making, regarding whether intervention appears justified, which intervention would be optimal (surgical revision or balloon angioplasty) and the specific method of intervention. Our perceived advantage of non-invasive imaging, in this way, rather than initial hemodynamic investigation in the catheterization laboratory, is that we achieve an, often crucial, "window" for discussion and procedure planning.

The potential vascular complications of catheterization [25,26], and the dangers of exposure to radiation [27] mean that for many centers, cardiac catheterization is reserved for patients in whom hemodynamic data is essential (e.g. high risk Fontan, pulmonary hypertension), or in whom it is known that interventional procedures are highly likely and necessary.

Finally, some patients benefits from a combined approach using a hybrid CMR/cardiac catheterization laboratory, in which patients can be transferred, under the same general anesthetic, from imaging to interventional procedures and vice versa. This guides the intervention procedure, and gives potential to immediately assess the hemodynamic results of intervention with assessment of flow and ventricular function [28,29] (see section 6).

## 3. Scanning environment, sequences and protocols

### 3.1. Scanning environment for general anesthetic cases (Figure 1)

Performing general anesthesia (GA) in a magnetic resonance environment is challenging for many reasons: [30] There is limited access to the child and ventilation equipment during the CMR scan; care is required for staff and patient safety with regards to ferromagnetic equipment; and there is a potential for RF interference with monitoring. It is therefore very important to have an appropriately trained anesthetic team (the cardiothoracic operative team in our institution), with excellent monitoring equipment. Several technical factors specific to MR in infants and small children must be taken into consideration. Prolonged, multiple breath holds are required, thus adequate pauses for ventilation control between breath holds are required, to ensure that hypoxia and hypercapnoea are avoided. Reliable monitoring of the electrocardiogram, pulse oximetry and expired gas concentrations is necessary. Additionally, patient temperature must be closely monitored. The low ambient temperature in MR scanning room produces a risk of hypothermia, particularly for small infants.

### 3.2 Sequences

Additional file 3, Table S3 describes the sequences that can be used for assessing patients with CHD. In Additional file 4, Table S4 suggestions are given for which sequences are most useful for a range of clinical indications.

Although time consuming, a full scanning protocol, including 3D data acquisition, is necessary for most patients because their complexity brings a high likelihood of previously undiagnosed or unexpected morphological or physiological findings. Acquiring a complete image data set gives the opportunity for full delineation of the sequential segmental anatomy in every patient.

#### 3.2.1. 3D imaging

The 3D capabilities of CMR play a key role for pediatric CHD. There are two conventional methods of acquiring 3D data. One uses angiographic techniques with gadolinium-based contrast agents that can be injected via any peripheral vein [31]. The other uses a 3D balanced-SSFP sequence, which is respiratory and cardiac gated, but does not require contrast [32,33]. Both data sets are acquired in such a way to give isotropic voxels, so that the images can be viewed with the same spatial resolution in any anatomical plane. These data can be used during the scan to plan image planes for further scanning, as well as during the reporting phase to assess 3D relationships between structures, quantify vessel size and view morphology. The high-signal, isotropic 3D images that are achieved using gadolinium-contrast angiography allow complex modeling of structures so that interventional techniques can be optimized [34].

#### 3.2.2. Cine imaging

Cine imaging using balanced-steady state free precession or fast gradient echo sequences, gives multiphase data that shows myocardial or valvular motion over the entire cardiac cycle. These cines have up to 40 frames per cardiac cycle, a temporal resolution adequate for accurate physiological representation. Cines can be performed in any plane to assess the dynamic function of any structure, including the outflow tracts, valves and great arteries. Furthermore, short-axis cine images, acquired in equal-width slices, perpendicular to the long-axis of the heart from base to apex (short axis imaging), or similar long-axis imaging in an axial plane, can be used to accurately assess cardiac function and measure the ventricular volumes.

The post-processing of cine images to calculate ventricular volumes and function is performed off-line, using commercially available software. The segmentation of the blood pool and myocardial border can be performed manually, or by using automated signal thresh-holding techniques. There is currently a wide range of software available, and a wide variation in segmenting practice and procedures. A fundamental issue, particularly for pediatric patients and those with congenital disease, is that of inclusion or non-inclusion of the trabeculae in the blood pool. If a simple endocardial contour is drawn and the trabeculae ignored and included in the blood pool, the manual segmentation process is more efficient and more reproducible [35]. However, this leads to erroneously large volume estimates for the ventricles, and prohibits internal validation of stroke volumes using great arterial flow volumes. Additionally, this could lead to the miscalculation of atrio-ventricular valve regurgitation.

#### 3.2.3. Flow assessment

Accurate quantification of flow volume is crucial in patients with known or suspected CHD. For volume quantification, we favor a free-breathing, velocity encoded, phase-contrast sequence with a temporal resolution of at least 30 frames per cardiac cycle. Slice positioning and velocity encoding must be optimized [36]. If these parameters are rigorously controlled, flow can be assessed in large and small arteries, systemic and pulmonary veins [10,37]. Aortic and pulmonary valve regurgitant fractions can be calculated. Phase contrast flow sequences also enable the profiling of flow acceleration jets, with velocity estimation. More importantly, with appropriate combinations of arterial and venous flow volume assessment, the technique allows accurate assessment of inter-atrial, inter-ventricular, arterial and venous shunt volumes. In the context of atrio-ventricular valve regurgitation, knowledge of the ventricular stroke volume, combined with knowledge of the forward arterial flow volume from that ventricle allows for calculation of mitral or tricuspid valve regurgitant fraction. For every patient in whom ventricular function is quantified, the practice of our unit is to undertake great arterial flow volume assessment to guide the volumetric analysis. This greatly enhances the accuracy and reproducibility of our reporting procedure [38].

#### 3.2.4 Black-blood Imaging

Spin echo pulse sequences can still play a role in imaging CHD. These sequences are effective for the assessment of the 2D morphology of the blood vessels and cardiac chambers, [39,40]. This is particularly useful when turbulent flow at the site of stenosis reduces the accuracy of balanced-SSFP or MRA images. Black blood imaging is also useful for elucidating the relationship between airway and blood vessels. This helps in identifying airway abnormality associated with various airway diseases, or in airway problems occurring as a complication of CHD [41]. Black-blood imaging is also useful when tissue characterization is necessary, in particular when fat infiltration of the myocardium is suspected. Though black-blood imaging has been suggested as a good method for assessing stents, we believe that this can give false re-assurance about stent patency (non-visualization of the stent interior) and hence we recommend other MR techniques to define stent morphology using CMR. These include using high-flip-angle gradient echo cine images, to assess the stent in longitudinal and cross-sectional planes [42].

#### 3.2.5. Late-gadolinium enhancement (LGE)

LGE-CMR has become an integral part of imaging both congenital and acquired cardiovascular diseases. This is achieved through the use of gadolinium-based agent and specific MR pulse sequences that help to differentiate between the normal and the diseased myocardium. The role of LGE-CMR in adults with ischemic cardiomyopathy has long been established [43], and its impact in the imaging work-up in pediatric population is growing. For patients with previously repaired tetralogy of Fallot, LGE has been associated with RV dilatation and worsening hemodynamics [44,45]. LGE has also been shown to be a good indicator of systemic RV failure in patients following atrial switch repair of transposition of great arteries [46]. Late after Fontan operation it has also been shown that LGE is associated with dilated and hypertrophied systemic ventricles, systolic dysfunction, regional dyskinesis and ventricular arrhythmias [47]. Areas of myocardial fibrosis following coronary artery re-implantation during repair of congenital heart diseases are also detected with LGE-CMR [48]. Moreover, the extent of late enhancement is associated with increased risk of arrhythmias and sudden death in adult patients with hypertrophic cardiomyopathy [49]. LGE-CMR has been shown to have a high diagnostic accuracy in patients with acute myocarditis [50].

#### 3.2.6. Stress perfusion CMR - adenosine and dobutamine

Myocardial perfusion CMR can be performed at rest and during stress with coronary vasodilatation induced by adenosine. This defines myocardial viability and the stress/rest adenosine perfusion deficit, while a bolus of gadolinium contrast agent is being administered. Indications for pharmacological perfusion CMR in the pediatric age group include suspected ischemia secondary to acquired coronary artery disease, such as Kawasaki's, or suspected ischemia following surgical transfer of coronary arteries during repair of CHD. The clinical value of adenosine perfusion CMR is similar to that of myocardial scintigraphy, with an advantage that adenosine perfusion is performed over a single 45-minute session, with no radiation exposure, as compared to two long sessions of scintigraphy. The high specificity and sensitivity of adenosine perfusion studies have been validated in adult patients with coronary artery disease [51,52].

There is very little data regarding the use of dobutamine stress CMR in pediatric patients with congenital disease. Currently our unit does not use this methodology, but many centres are gathering experience. The feasibility has been shown in one small study [53]. Some centres are utilizing dobutamine stress methodology for additional decision support in the decisions regarding timing for intervention, for example in the population of patients with repaired tetralogy of Fallot [54,55].

#### 3.2.7. Sequence optimization

Pediatric CMR poses various technical challenges that need to be considered in order to obtain optimal images to answer the clinical question being investigated. These include: fast heart rate in neonates and infants (100-150 beats per minute) requiring a high temporal resolution for accurate ventricular volume and flow measurements; small-sized heart and blood vessels requiring greater spatial resolution;[56,57] and potential arrhythmias as complications surgical procedure or the congenital anomaly itself. These will render CMR difficult, and will require adjustments to normal CMR imaging protocols.

To meet the needs of successful pediatric CMR, some adjustments are as follows. Due to small size of the heart and blood vessels, slice thickness is reduced to 3-5 mm. The field of view is also reduced, but this is at the expense of signal:noise ratio (SNR). Sometimes, smaller size, pediatric radiofrequency coils or the application of multiple signal averages can help to maintain the SNR at high spatial-resolution for these small hearts. To avoid image blurring due to fast heart rate in newborns and infants, it is necessary to improve the temporal resolution. Reducing the number of views to be acquired per segment in the segmented k-space and minimizing repetition time (TR) during each cardiac cycle in retrospective sequences such as balanced SSFP cine, will help improve the temporal resolution. In prospectively gated sequences such as turbo spin echo (TSE) every other heart beat techniques can be applied [57-59]. In patients with arrhythmias, real time imaging can be used [60,61]. In patients having difficulty with breath-holding, or with respiratory motion artifacts, averaging techniques or a respiratory navigator can be applied.

#### 3.2.8. A note on normal values in children

The rapid uptake of CMR and exponential rise in use for pediatric cardiology accentuates the paucity of CMR data giving normal reference values for pediatric patients. Normal data for ventricular volumes, function and other structural measurements has been published [19,62,63] and multicentre data is now being accumulated. It is crucial that these data incorporate, or at least attempt to unify, the multitude of different imaging and post-processing conventions that have evolved in the international centers developing pediatric CMR.

## 4. Clinical applications

### 4.1. Cardiovascular shunts

The suspicion of significant systemic to pulmonary shunt at any level; intrapulmonary, atrial, ventricular or systemic arterial, can be an indication for CMR, with the aim to assess the anatomy, quantify the shunt, and measure the effect of any volume loading on the atrial and ventricular chambers.

Conventionally, the most common methods of evaluation of these shunts have been invasive oximetry or thermal dilution, non-invasive first-pass radionuclide angiography or color Doppler echocardiography. All of these techniques have significant limitations [64]. CMR techniques have been shown to correlate well with oximetry and Doppler echocardiography in quantification of the shunt volume [65-67].

Importantly, CMR also gives morphological information to guide intervention and management. Gaps in septal signal in dark-blood acquisitions may suggest the presence of defects, but this may be due to partial volume effects and possible signal drop out [65]. Suspected defects should be investigated further by appropriately aligned cine and velocity map acquisitions [68]. Signal artefact caused by flow turbulence through the defect can be visualized during different phases of the cardiac cycle by white-blood cine imaging techniques such as SSFP, orientated perpendicular to the adjacent septum, and acquired in stacks of relatively thin slices, without gaps. This can be followed by a through-plane or in-plane flow velocity acquisition, transecting the jet emerging through the defect [69], prior to flow velocity mapping within the great vessels [70] to quantify the shunt volume.

Quantification of the left to right shunt is traditionally based on Flick's principle, which looks at the ratio of pulmonary (Qp) to systemic (Qs) flow. The feasibility of CMR to quantify intra-cardiac shunt has been shown to correlate well with the other methods. Quantification of blood flow is done using velocity encoded cine CMR (VENC-CMR), carefully optimized for spatial and temporal resolution, and planned in a plane perpendicular to the direction of flow in the relevant great vessels. At the simplest level, by measuring the flow volume in both the main pulmonary artery and the proximal ascending aorta, a Qp/Qs ratio is obtained [11,70-74]. Correlating this data with ventricular stroke volumes, can give the level of the shunt.

### 4.2. Diseases of the aorta

**4.2.1. Coarctation of the aorta (CoA) **is a congenital narrowing of the aorta, usually at the site of ductal insertion (aortic isthmus) [75]. The treatment of choice in infancy is surgery, though in older subjects balloon angioplasty or stent implantation can give effective relief of arch stenosis.

**Figure 2:**
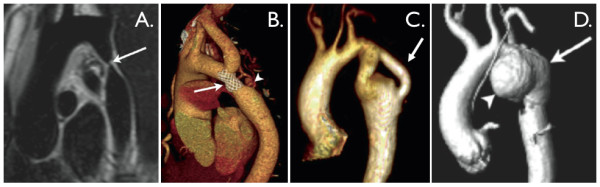
**Aortic coarctation**. **A**. 'Black-blood' oblique sagittal view showing discrete, tight coarctation at the aortic isthmus (arrow). **B**. 3D, contrast-enhanced CT angiogram showing mildly narrowed bare metal stent (arrow) that partially overlies the left subclavian artery origin. The arrowhead shows a subtle pseudo-aneurysm at the distal end of the stent. **C**. 3D, contrast-enhanced MR angiogram showing aortic arch hypoplasia and coarctation with a 'jump' by-pass graft posteriorly (arrow). **D**. 3D, contrast-enhanced MR angiogram showing large pseudo-aneurysm (arrowhead) after previous patch angioplasty repair. The true lumen is shown posteriorly (arrow).

CMR is the first line assessment in the follow-up of CoA (Figure 2), and can identify the arch geometry and morphology (residual stenosis or aneurysm formation), as well as assess aortic valvular morphology, and left ventricular systolic function and hypertrophy. A "gothic" arch is associated with high risk of resting hypertension despite successful repair [76]. CMR can characterise coarctation stents, using black blood and gradient echo cine sequences. Stent-associated stenosis can also be diagnosed with phase contrast flow mapping and angiography, or high-flip angle gradient echo cine images [42]. However, often cardiovascular CT may also help to assess internal stent morphology and adjacent complications.

**4.2.2. Interrupted aortic arch (IAA) **is rarely imaged pre-operatively in the neonate with CMR, as echocardiography can usually define the arch anatomy and associated intracardiac anomalies [77]. Post-operative CMR imaging has the same advantages as for simple coarcatation aorta, and the imaging protocols used generally correlate.

**4.2.3. Anomalies of the aortic arch **are due to failure of fusion and regression of the brachial arches in a usual manner during the embryologic development of the aortic arch, pulmonary arteries and ductus arteriosus [78,79]. The diagnosis of these abnormalities using CMR can be achieved by contrast enhanced (CE-MRA) and non-contrast enhanced 3D SSFP sequences, which delineate the anatomy very well, [80] and can often depict associated airway anomalies.

### 4.3. Disease that predominantly affect the right ventricle

**4.3.1. Tetralogy of Fallot **is the most common cyanotic congenital heart disease accounting for 420 per million live births [1]. CMR has become a prominent diagnostic and monitoring tool for both pre- and post- operative assessment of tetralogy of Fallot [81-83].

**Figure 3:**
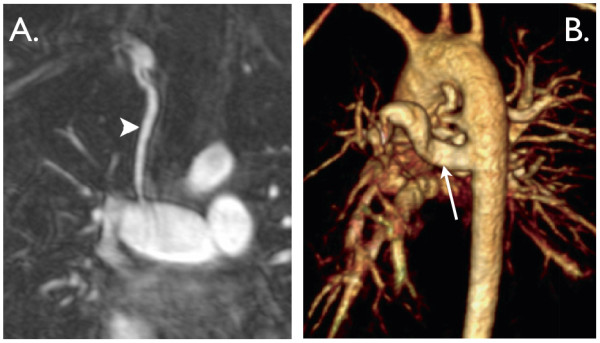
**A. Coronal view from a contrast-enhanced MR angiogram showing a modified BT shunt (arrowhead)**. It originates from the innominate artery and inserts into a dilated right pulmonary artery. **B**. 3D, contrast-enhanced MR angiogram viewed from left posterior lateral showing several major aorto-pulmonary collateral arteries (MAPCAs). The arrow shows the largest MAPCA to the right lung.

Echocardiography is usually sufficient to define anatomy prior to surgery in most patients during infancy, however those with complex pulmonary stenosis or atresia can be effectively assessed with CMR [84], with the aim of identifying the presence and the size of the native pulmonary arteries and the source of pulmonary blood supply (Figure 3). CMR defines the degree of RVOT obstruction [40,85,86], and with the use of 3D SSFP sequences, can define the coronary anatomy, to exclude the presence of a large coronary artery branch crossing the RV outflow tract.

**Figure 4:**
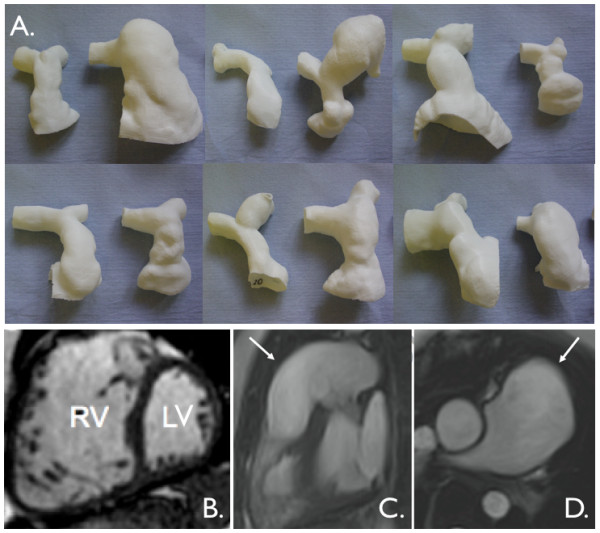
**Repaired tetralogy of Fallot**. **A**. 3D rapid prototyping models of the right ventricular outflow tract, pulmonary trunk and branch pulmonary arteries (reconstructed from 3D, contrast-enhanced MR angiogram data) from 12 patients with tetralogy of Fallot, all repaired in infancy and imaged 12-15 years later. Note the wide variation in morphology, size and narrowings. **B**. End-diastolic, balanced-SSFP, mid-ventricular, short-axis view showing severely dilated right ventricle (RV), flattened septum and small left ventricle (LV). **C**. (sagittal) &**D**. (axial), end-systolic, balanced-SSFP images of an aneurismal right ventricular outflow tract (arrow).

The degree of RV dilatation secondary to chronic volume load, as a result of pulmonary regurgitation, has a deleterious impact on biventricular systolic function and functional efficiency [87-89] (Figure 4). Currently the main treatment of severe pulmonary regurgitation in this population is the replacement of the pulmonary valve. This can be achieved surgically or trans-catheter percutaneous pulmonary valve implantation  (PPVI). CMR provides a basis for deciding which route to employ in replacing the pulmonary valve and has demonstrated significant physiological improvement following PPVI [90,91].

Pulmonary stenosis following Tetralogy repair can be well characterised by CMR, using cine imaging and flow mapping. PC-MRI is the best modality in demonstrating the relative volume of blood flow to each lung after the repair of Tetralogy of Fallot [92]. CMR has been shown to be sensitive and specific in detecting branch pulmonary artery stenosis following Tetralogy repair especially 3-D MRA [93-95]. RV diastolic function can be assessed with tricuspid valve inflow volumetric curves using PC-MRI [96].

**4.3.2. Transposition of the great arteries (TGA) **comprises 3% of all congenital heart disease [1]. CMR is seldom required for pre-operative assessment of simple TGA, as echocardiography usually provides adequate diagnostic information [97]. The main indication for CMR in TGA is the evaluation of post-operative complications.

Surgical therapy for this condition was revolutionized in the 1960's with the introduction of the Senning and the Mustard procedures (atrial switch operations), which involved the diversion of systemic venous return to the left ventricle and pulmonary venous return to the right ventricle. This creates a physiological correction of the problem with a very abnormal anatomy.

**Figure 5:**
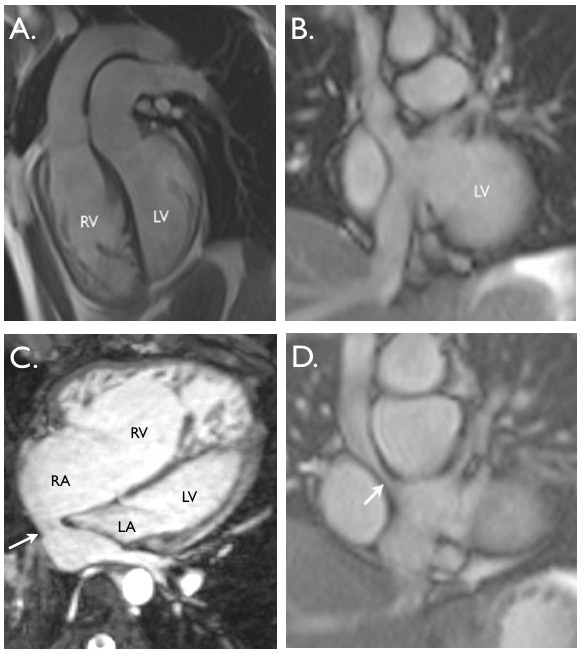
**Transposition of the great arteries - Atrial switch (Senning or Mustard) operation**. All images taken from frames of balanced-SSFP data. A. Oblique sagittal view through the ventricular outflow tracts showing the aorta arising anteriorly from the right ventricle (RV) and the pulmonary trunk posteriorly form the left ventricle (LV). B. Oblique coronal view through the systemic venous baffle, with both the SVC and IVC directed to the left atrium and then to the LV. C. Oblique axial view showing the pulmonary venous baffle (arrow) connecting the pulmonary veins to the right atrium and then RV. D. Oblique coronal view showing SVC baffle narrowing (arrow).

Post-atrial switch assessment involves cine and 3D imaging of the venous pathways for baffle leaks or obstruction, and assessing systemic RV systolic function and tricuspid valve function (Figure 5). Late gadolinium enhancement of the ventricular myocardium after atrial switch operation has been found to correlate with outcome [98,99].

In the current era, the surgical procedure of choice for neonates diagnosed with TGA is the arterial switch operation [100,101]. This produces both physiological and anatomical correction.

**Figure 6:**
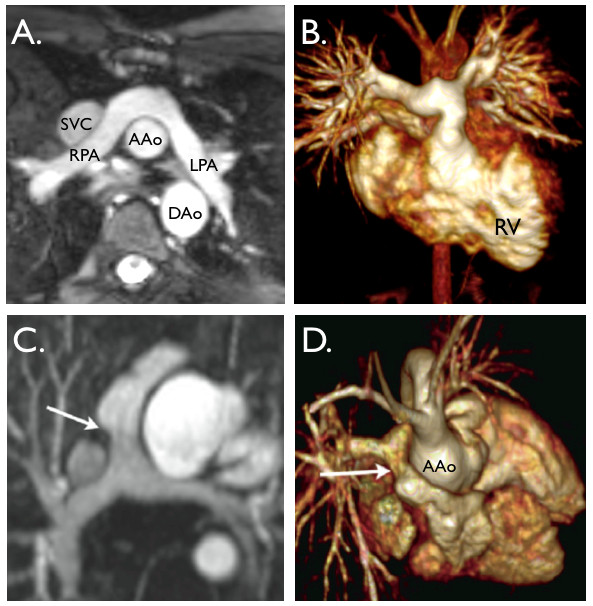
**Transposition of the great arteries - Arterial switch operation**. **A.** Axial reformat from contrast-enhanced MR angiogram &B. 3D, contrast-enhanced MR angiogram. Both **A** and **B** show Lecompte maneuver with the pulmonary artery anterior to the ascending aorta (AAo) with the right (RPA) and left pulmonary arteries passing either side of the aorta, note descending aorta (DAo). **C**. (axial reformat from contrast-enhanced MR angiogram) &**D**. (3D, contrast-enhanced MR angiogram) Showing alternative arterial switch operation, with the main pulmonary artery (arrow) seen to pass on the right side, between the superior vena cava (SVC), and aorta.

Although the arterial switch operation (ASO) has excellent long-term outcomes, there can be serious complications concerns related to this surgical procedure. The main complications of ASO are main pulmonary artery or branch pulmonary artery stenosis, related to the LeCompte maneuver [102] (Figure 6). Additionally, dilatation of the neo-aortic root and regurgitation of the neo-aortic valve can cause hemodynamic complications in the long term. Assessment of these post operative complications involve CMR techniques previously described in this review; stenosis, valve function and baffle leaks are assessed using PC-MRI and anatomical and physiological assessment employs the use of spin echo, gradient echo and 3-D MRA [103,104].

Proximal coronary artery geometry can be assessed by MRA or MDCT and the presence of reversible damage caused by the stenosis is assessed by pharmacologically induced myocardial perfusion stress using adenosine or dobutamine [48,51,105].

**4.3.3. Double outlet right ventricle (DORV) **is a rare cyanotic congenital heart malformation in which both great arteries arise predominantly from the right ventricle. There is almost always a VSD that acts as an outlet from the left ventricle. DORV is classified according to the relationship (commitment) of the VSD to each of the great vessel's valve and the most common sub-groups are:

• Sub-aortic VSD (Fallot physiology) has pulmonary stenosis and if there is no associated pulmonary stenosis it presents with VSD physiology. This is the most common type of DORV.

• Sub-pulmonic VSD with transposition of the great vessels (Taussig-Bing type) DORV [86].

• Double committed: where the VSD is committed to both great arteries.

• Non-committed VSD

The ultimate goal of management of these patients is to align LV with systemic outflow tract and RV with the pulmonary outflow tract. The LV can be hypoplastic, and these are the groups that pose challenges to the surgeon. Detailed imaging is therefore mandatory to assess the anatomical relations and ventricular physiology before deciding surgical strategy for a biventricular or single ventricular repair. CMR has replaced invasive cardiac catheterization for this cause and has been shown to correlate very well with surgical findings in investigating the exact position of great arteries and their relationship to the VSD [106,107]. The 3-D isotropic CMR is ideal for assessing this complex type of anatomy. Post-operative complications are usually imaged using a combination of black-blood images, balanced-SSFP, PC-CMR and 3-D MRA. With the tetralogy physiology complications are similar to those experienced with tetralogy of Fallot, and with the Taussig-Bing type complications are similar to those experienced in TGA (see previous sections).

### 4.4. Complex congenital heart disease

**4.4.1 The single ventricle heart **is a complex entity that encompasses varying degrees of anatomic and physiologic states in which only one ventricle supports the circulation. Specific anatomical examples include hypoplastic left heart syndrome (HLHS), which is characterized by the underdevelopment of the left-sided heart structures, and tricuspid atresia (hypoplastic right heart).

**Figure 7:**
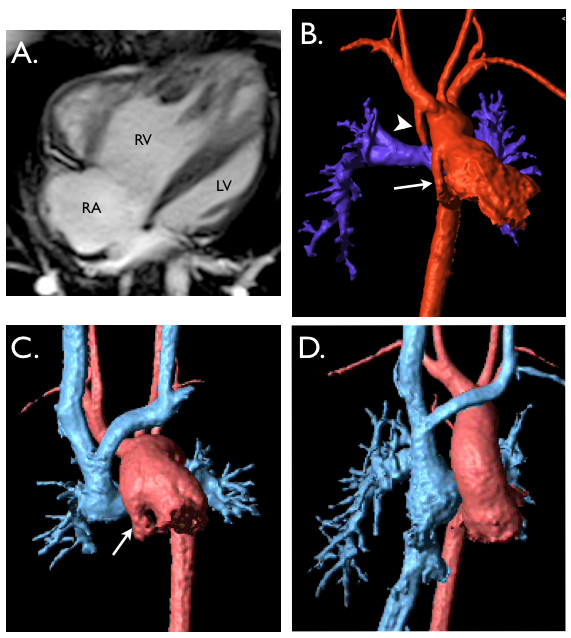
**Hypoplastic left heart syndrome**. **A**. End-diastolic, balanced SSFP, 4 chamber-view showing hypoplastic left ventricle (LV). Pulmonary venous return passes from left atrium to the right atrium, via a large atrial septostomy. **B**. 3D, contrast-enhanced MR angiogram after Stage 1, Norward operation, with a modified BT shunt (arrowhead) supplying the pulmonary arteries [153]. Note the hypoplastic native ascending aorta (arrow). **C**. 3D, contrast-enhanced MR angiogram after Stage 2 bi-directional cavo-pulmonary connection operation. This connects the SVC to the branch pulmonary arteries (pale blue). Again arrow shows hypoplastic native ascending aorta. **D**. 3D, contrast-enhanced MR angiogram after Stage 3, total cavo-pulmonary connection operation. This further connects the IVC into the pulmonary circulation (pale blue).

For simplicity we will use HLHS as our reference point to illustrate imaging issues of the uni-ventricular heart. Management of a single ventricle involves a series of staged palliative procedures. CMR can valuably contribute intervention planning before and after each stage of the palliative surgical process, and keeps radiation exposure at a minimum (Figure 7). CMR has been shown to be superior to both x-ray angiography and echocardiography in this group of patients [17,108-110].

Prior to any procedure, a number of centres are using CMR methodology to give decision support for patients with "borderline size" ventricles. For those within the spectrum of hypoplasia of the left ventricle, LGE techniques can often identify significant endocardial fibroelastosis [111]. Long-term outcome data for patients studied and classified with these techniques remains under investigation.

After the first stage of surgery, CMR, using black-blood sequences or gadolinium-enhanced angiography can delineate the aortic arch and branch pulmonary artery anatomy, and visualize the aorto-pulmonary shunt. The second surgical stage is most frequently the bidirectional superior cavopulmonary connection (BCPC) at age 4-6 months. There are various aspects of the haemodynamics that need to be considered when assessing a patient post-BCPC and before the final stage of palliation - formation of the total cavopulmonary circulation. These include the ventricular function, the aortic arch for obstruction, the caliber and patency of the branch pulmonary arteries, shunting through the collateral vessels, and adequacy of inter-atrial communication [112]. The BCPC is a low flow velocity circuit and its CMR is achieved using cine, contrast enhanced and PC-CMR sequences. Assessment of a BCPC circuit non-invasively for collaterals using the PC-CMR is reliable [113].

The final stage of a single ventricular repair is the creation of a Fontan-type circuit in which the SVC and IVC blood is directed into the pulmonary arteries and completely bypasses the heart to enter the lungs. CMR presents valuable 3D morphological and functional information regarding the Fontan circulation, as well as the possibility to sensitively assess for thrombus.

**4.4.2. Defining atrial morphology and associated findings**: Abnormal atrial situs is often associated with complex cardiac malformations and abnormal abdominal and thoracic anatomy [86]. 3D balanced-SSFP images are valuable for determining the atrial situs. Isomerism of the right atrial appendages is associated with bilateral right bronchi and tri-lobed lungs, bilateral right atrial appendages, asplenia and midline liver. The left atrial isomerism is associated with bilateral left bronchi; bi-lobed lung, bilateral left atrial appendages, polysplenia and interrupted IVC [114,115].

### 4.5. Assessment of coronary artery problems

Congenital coronary artery anomalies are rare, affecting 0.3-0.8% of the population [116]. CMR is a valuable adjunct for the assessment of anomalous coronary arteries [117,118]. 3D mapping of the coronary morphology using respiratory and cardiac-gated balanced SSFP imaging can reveal the proximal course of the coronary arteries and delineate aneurismal dilatation. The definition of the proximal course of the coronary arteries is becoming increasingly important in the assessment of patients who are undergoing interventions to cardiac structures in close proximity to the coronary arteries for example percutaneous pulmonary valve implantation into the pulmonary trunk [119], or stenting of the branch pulmonary arteries in ASO.

Multi slice CT coronary angiography has been shown to have sensitivity, specificity and negative predictive value of almost 100% in assessing coronary artery problems following ASO for TGA [120].

Though CMR does not portray lumen patency well, CMR myocardial stress perfusion and late gadolinium studies are the gold standard for assessment of end-organ function in adults: characterizing myocardial ischemia, scarring and viability respectively [44,46,121]. There are many pediatric and adolescent populations with congenital heart disease (post arterial switch operation [48], post Kawasaki's disease, post coronary re-positioning surgery) who may benefit from assessment of coronary adequacy. Many centers are exploring this, and finding success in pediatric patients [53,122].

## 5. Emerging indications

There are other patient groups in which the benefits of and indications for CMR are well validated in the adult population, but where there is currently a paucity of data pertaining to patients in the pediatric age range. Many factors limit the comparability of adult and pediatric populations, however the potential for pediatric CMR in these fields is rapidly being realized.

### 5.1. Cardiomyopathy assessment

**Figure 8:**
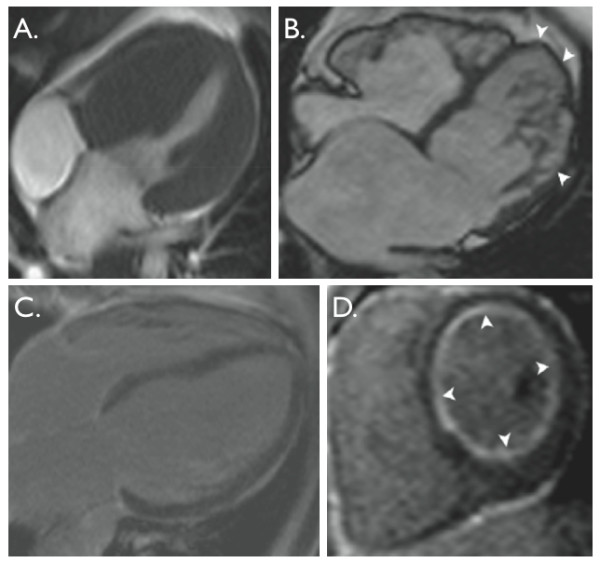
**Examples of cardiomyopathies**. **A**. 4-chamber, balanced-SSFP view in hypertrophic cardiomyopathy. Note the marked thickening of the septum with compression of the RV cavity. **B**. 4-chamber, balanced-SSFP view in left ventricular non-compaction. Note the arrowheads show areas of thin compacted myocardium. **C**. 4-chamber, late gadolinium enhancement (LGE) image in idiopathic dilated cardiomyopathy. Note no LGE. **D**. Short-axis, LGE image in a patient with critical aortic stenosis, restrictive cardiomyopathy secondary to global, sub-endocardial fibrosis.

CMR is showing great potential in the pediatric population for the diagnostic assessment and therapeutic monitoring of patients with all types of cardiomyopathies [123,124] (Figure 8). CMR has the capacity to acquire images without acoustic limitations, in 3-dimensions, with tissue contrast and myocardial border definition that is often superior to echocardiography. This gives great advantage for pre-clinical diagnosis or family screening [125,126]. CMR has the advantage of accurate quantification of segmental function, ventricular volume and systolic shortening, while sensitively imaging myocardial architecture. The extent of LGE in patients with HCM has been independently associated with adverse outcome and worsening clinical symptoms, suggesting its link to prognosis and its ability to be used as an independent risk factor in these patients [127,128]. This comprehensive assessment also enlightens clinical and pharmacological management in the many types of dilated cardiomyopathies and skeletal myopathies with cardiac involvement [129].

### 5.2. Iron loading

Cardiac T2* assessments for myocardial iron loading [130] are an increasing referral source for CMR assessment. Pediatric patients with thalassemia major, or other chronic anemias requiring multiple transfusions are at risk of myocardial iron deposition, progressive fibrosis and systolic impairment. The optimal timing for screening of these young patients by CMR is under debate. Some evidence suggests that initiation of assessment should be determined according to the patient's age and transfusional burden [131]. When the appropriate chelation therapy has been administered since birth, CMR can be postponed until 8 years of age, so that anesthesia is not required for the scan. Patients with suboptimal chelation or with increased transfusional requirements should be tested sooner. However, as with many other pediatric pathologies, the CMR T2* technique for iron assessment has only been validated in adults. No validation or range of normal values exists for the infant and pediatric population.

## 6. Role of the hybrid CMR/catheter laboratory

**Figure 9:**
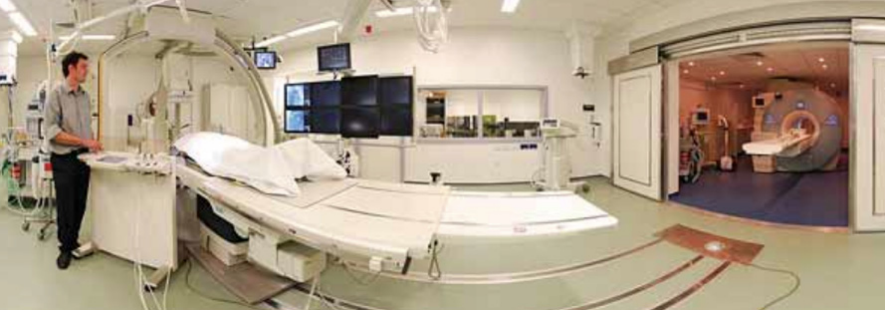
**Hybrid CMR/cardiac catheterization laboratory**. Fish-eye view of a hybrid CMR/cardiac catheterization lab - The bi-plane catheter lab (left) is connected to the MR scanner room (right), via a set of sliding doors (open). The pedestal of the catheter table slides toward the MR scanning room to join with the MR scanner table. The patient then slides across between the two tabletops, using Miyabi table technology (Siemens AG).

The hybrid MR/X-ray catheter suite (XMR) is emerging as a useful diagnostic and interventional tool for cardiovascular diseases in both children and adults (Figure 9). There are many attractive attributes to these hybrid suites as compared to purely X-ray techniques, which have been the gold standard imaging modalities in cardiovascular medicine.

XMR reduces the amount of radiation exposure to both patients and medical staff due to the lack of ionising radiation of the MR imaging component [132,133]. This is mainly important for children who are prone to DNA and chromosomal damage by radiation exposure leading to development of malignancies [134,135].

CMR provides a detailed anatomy, which is useful in pre-procedural planning for electrophysiological studies and cardiac interventional procedures. In electrophysiology, CMR helps to identify the scar tissue acting as the focus for the abnormal electrical impulse and it also gives a detailed anatomy of adjacent structures to prevent ablation therapy related complications [132,136,137]. Various studies have shown the feasibility of CMR guided intervention due to its superior soft tissue quality and having an XMR means the X-ray can be used as a bail out if need arise [138-142].

CMR use in physiological studies such as pulmonary vascular resistance (PVR) and left and right heart catheterization seems to be coming out of its shell. In an XMR suite both invasive pressure data and flow data can be acquired. This is particularly good in PVR studies and in quantification of collateral flow in cavo-pulmonary connection patients including ventricular function assessment [17,29,133,143,144].

## 7. Role of cardiovascular CT

CT imaging also plays an important role in the management of pediatric CHD. This modality provides very high-resolution 3D data sets with an extremely short acquisition period and therefore can usually be performed in infants and small children without general anesthetic. The expense of this imaging is the exposure of patients to potentially large doses of ionizing radiation, particularly for ECG-gated studies, though this continues to fall. Its use for serial evaluations is therefore very limited. CT imaging is useful for patients who are unable to co-operate with CMR or who are too clinically unstable to undergo general anesthetic. Additionally, when CMR provides inadequate images for clinical decision-making, CT angiography is the modality of choice in:

• Patients with vascular rings, where it is important to visualize the airway anatomy.

• Patients in whom we are investigating pulmonary venous anatomy (in our experience MR imaging of the pulmonary veins can be problematic).

• Patients in whom we are assessing pulmonary atresia with major aorta pulmonary collateral arteries (MAPCAs) - our protocol for assessing these patients is to perform a CT scan prior to cardiac catheterization. The CT scan will identify the number of large aorta pulmonary collaterals and the presence of any central pulmonary arteries, and this information can be used to guide cardiac catheterization. The main purpose of the cardiac catheterization is to identify the temporal distribution of blood flow and define which areas of the lungs the pulmonary arteries, the MAPCAs, or both supply. This significantly aids the surgeons in unifocalisation in these patients.

• Patients who have metallic implants - e.g. routine CT following aortic coarctation stenting at 3 months to exclude pseudo-aneurysm formation at the distal ends of the stent.

• Patients in whom there is contraindication to CMR (e.g. permanent pacemaker).

## 8. Future directions and conclusions

### 8.1. Real-time imaging

This technique employs continuous imaging of dynamic cardiovascular processes in real time using (SENSE, SMASH and their variants). Data acquisition is accelerated for any CMR pulse sequence with the use of parallel imaging. Importantly, there is no need of cardiac gating and breath holding, which is an added advantage for an uncooperative and poor breath-holder pediatric patient [145,146]. The fact that it is real-time means that it is very useful in interventional CMR with endovascular devices and in CMR guided catheterization where position of catheters or devices can be tracked in real-time.

### 8.2. 4D flow

Time resolved 3D (4D) phase contrast flow velocity acquisition allows the reconstruction of multidirectional flow velocities; measurements for each phase of the cycle being effectively averaged over many heart cycles. Such acquisitions typically take 10 minutes or more, so beat-to-beat variations related to respiration or flow instabilities are not represented. Besides the visualization of principal multidirectional flow paths, this offers the potential to retrospectively quantify flow through selected planes in the volume covered [147]. Reported applications include the depiction of large-scale flow patterns in the aortas of patients with bicuspid aortic valves and the retrospective measurements of flow in the presence of more than one shunt [148,149]. Moreover, this method has also been used in the evaluation of Fontan pathway dynamics [150,151].

### 8.3. Exercise

**Figure 10:**
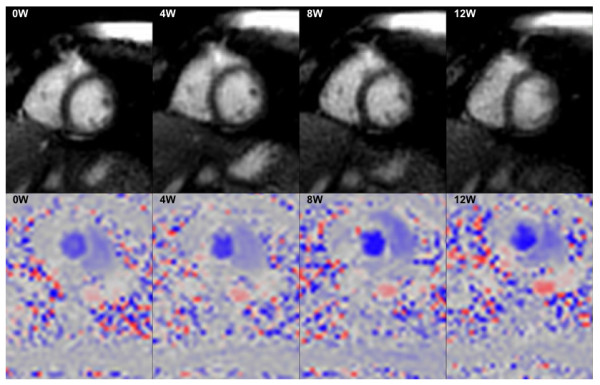
**Real time data**. Short-axis ventricular volumes (top) and flow data (bottom) acquired during increasing exercise within the MR scanner (0, 4, 8, 12W). Because the data is acquired in real-time, there is no need for the patient to attempt breath-holding during peak exercise, which is often difficult to achieve.

Progressive worsening of the symptoms related to cardiovascular disease can be masked at rest and only brought out through pharmacological stress or physical exercise. Physiological response seems to differ between pharmacological and exercise induced stress, with exercise more superior to pharmacological stress [152]. Real time biventricular volumetric assessment has been validated and found to be feasible and reproducible [145]. This helps to ensure that the physiological CMR changes secondary to exercise are acquired simultaneously, with the use of an MR compatible ergometer (Figure 10).

### 8.4. Conclusion

CMR is now a major imaging tool in pediatric congenital heart disease. It is made attractive by its non-invasiveness and lack of ionizing radiation. The technological advancements, with improved image resolution and ultra-short imaging time, have allowed real-time imaging to come to the fore. This lends towards the added advantage of CMR-guided catheterization and interventions. Promising studies done in this and the many other areas described in this review show that CMR will revolutionise pediatric cardiology practice due to the radiation-free environment it provides [153].

## Conflict of interests

The authors declare that they have no competing interests.

## Authors' contributions

HNN: Drafted the manuscript, reviewed literature, prepared the manuscript and approved the final version of this manuscript; MLH: Drafted the manuscript, reviewed literature, prepared the manuscript and approved the final version of this manuscript; AMT: Drafted the manuscript, reviewed literature, prepared the manuscript and approved the final version of this manuscript.

## Supplementary Material

Additional file 1 **Table S1**. Common indications for pediatric CMR under general anesthetic. Click here for file

Additional file 2 **Table S2**. Common indications for pediatric CMR without anesthetic (usually, children greater than 7 years age). Click here for file

Additional file 3 **Table S3**. Example of the standard sequences and views of a usual pediatric congenital cardiac scan, in the order of workflow. Click here for file

Additional file 4 **Table S4**. Sequences that are useful in various clinical conditions. Click here for file
