# Supplementary material for: The Role of Cardiovascular Magnetic Resonance in Pediatric Congenital Heart Disease
Source: J Cardiovasc Magn Reson. 2011 Sep 21;13(1):51. doi: 10.1186/1532-429X-13-51 (PMC3210092; doi:10.1186/1532-429X-13-51)
Supplement: Additional file 1 — Table S1. Common indications for pediatric CMR under general anesthetic. [file 1532-429X-13-51-S1.DOC]

**Table S1** – Common indications for pediatric CMR under general anesthetic

| **PATIENT GROUP** | **INFORMATION SOUGHT** |
| --- | --- |
| Aortic arch abnormalities  Coarctation  Interrupted arch  Pre & post repair  Vascular ring | Aortic arch morphology & dimensions  LV function & mass  LV outflow tract status  Aortic valve function and flow assessment |
| Aortic arch connective tissue disease | Aortic arch morphology & dimensions  Aortic dissection & compliance  LV volume, function & mass  LV outflow tract |
| Uni-ventricular heart pre-BCPC  (including Norwood I) | Pulmonary artery morphology  Aortic arch morphology  Ventricular volume & function  Valvular function & flow assessment |
| Uni-ventricular heart pre-TCPC  (including BCPC or hemi-Fontan) | Jugular venous pressure under GA  Pulmonary artery morphology  Aortic arch morphology  Ventricular volume & function  Valvular function with flow assessment  Quantification of collateral flow |
| Tetralogy of Fallot with BT shunt | RV outflow tract morphology  Pulmonary artery morphology |
| PA/VSD, DORV, Common arterial trunk | RV outflow tract morphology  Pulmonary artery morphology Aortopulmonary collateral arteries |
| TGA – post arterial switch assessment | RV outflow tract morphology  Pulmonary artery morphology  Aortic arch morphology  Ventricular volume & function  Valvular function with flow assessment  Ventricular scarring or fibrosis |

(Table 1 continued)

| Pulmonary vein abnormalities | Pulmonary vein morphology & flow  Pulmonary : systemic flow ratio |
| --- | --- |
| Cardiomyopathy (HCM, DCM) | Myocardial characterisation  Ventricular scarring or fibrosis  Ventricular function  Outflow tract obstruction  Valvular function & flow assessment |
| Kawasaki assessment | Coronary morphology  Ventricular scarring or fibrosis  Ventricular volume & function |
| Cardiac tumour | Tumour characterisation  Ventricular scarring or fibrosis  Ventricular volume & function  Outflow tract and valvular function |

(ASD: Atrial septal defect, VSD: Ventricular septal defect, PAPVD: Partial anomalous pulmonary venous drainage, PA: Pulmonary artery, CoA: Coarctation aorta, BCPC: Bidirectional cavo-pulmonary connection, TCPC: Total cavo-pulmonary connection, BT: Blalock-Taussig shunt, PA/VSD: Pulmonary atresia with ventricular septal defect, TGA: Transposition of the great arteries, HCM: Hypertrophic cardiomyopathy, DCM: Dilated cardiomyopathy, RV: Right ventricle)
