# Supplementary material for: The Role of Cardiovascular Magnetic Resonance in Pediatric Congenital Heart Disease
Source: J Cardiovasc Magn Reson. 2011 Sep 21;13(1):51. doi: 10.1186/1532-429X-13-51 (PMC3210092; doi:10.1186/1532-429X-13-51)
Supplement: Additional file 2 — Table S2. Common indications for pediatric CMR without anesthetic (usually, children greater than 7 years age). [file 1532-429X-13-51-S2.DOC]

**Table S2** – Common indications for pediatric CMR without anesthetic (usually, children greater than 7 years age)

| **PATIENT GROUP** | **INFORMATION SOUGHT** |
| --- | --- |
| Regurgitant valves | Outflow tract morphology  Valve morphology & function  Forward & regurgitant flow quantification  Ventricular volume & function |
| ASD, VSD shunt | Defect position & size  Net shunt quantification (arterial flow)  Ventricular volume & function |
| PAPVD | Pulmonary vein morphology  Net shunt quantification (arterial flow)  Ventricular volume & function |
| Pulmonary valve stenosis | RV outflow tract morphology  Pulmonary artery morphology  Valvular function with flow assessment  Ventricular volume & function |
| Branch PA stenosis | RV outflow tract morphology  Pulmonary artery morphology  Valvular function with flow assessment |
| Repaired Tetralogy of Fallot (including conduit patients – e.g. PA/VSD, post- Rastelli operation) | RV outflow tract morphology  Pulmonary artery morphology  Ventricular volume & function  Valvular function with flow assessment  Coronary artery position  Ventricular scarring or fibrosis |
| Repaired common arterial trunk | RV outflow tract morphology  Pulmonary artery morphology  Ventricular volume & function  Valvular function with flow assessment  Ventricular scarring or fibrosis |
| TGA – post atrial switch operation (Mustard and Senning operations) | Systemic RV volume & function  Atrial baffle morphology & function  Valvular function with flow assessment  Ventricular scarring or fibrosis |
| TGA - post arterial switch operation | RV outflow tract morphology  Pulmonary artery morphology  Aortic arch morphology  Ventricular volume & function  Valvular function with flow assessment  Coronary artery position  Ventricular scarring or fibrosis |
| Aortic arch: Native or repaired CoA | Aortic arch morphology & dimensions  LV function & mass  LV outflow tract status  Aortic valve function and flow assessment |
| Aortic arch: Marfan, connective tissue disease | Aortic arch morphology & dimensions  Aortic compliance, dissection  LV volume, function & mass  LV outflow tract. |
| Pulmonary vein stenosis – post repair of PAPVD or TAPVD | Pulmonary vein morphology & flow  Differential branch pulmonary artery flow  Pulmonary:systemic flow ratio |
| Uni-ventricular – post BCPC, hemi-Fontan | Pulmonary artery morphology  Aortic arch morphology  Ventricular volume & function  Valvular function with flow assessment  Quantification of collateral flow |
| Uni-ventricular - Fontan / TCPC | Fontan pathway morphology  Pulmonary artery morphology  Aortic arch morphology  Ventricular volume & function  Ventricular scarring or fibrosis  Valvular function with flow assessment  Quantification of collateral flow |
| Ebstein anomaly | Tricuspid valve leaflet morphology & function  Right atrial volume  RV volume & function  Quantification of net forward flow  Quantification of ASD shunt |

(Table 2 continued)

| Complex anatomy – any | 3D morphology: connections, outflow tracts  Pulmonary artery morphology  Aortic arch morphology  Ventricular volume & function  Valvular function with flow assessment  Quantification of net shunt |
| --- | --- |
| T2* | Ventricular volume and function  Myocardial iron loading status  Hepatic iron loading status |
| Cardiomyopathy  (DCM, HCM, ARVC, skeletal myopathies) | Myocardial characterisation  Ventricular scarring or fibrosis  Ventricular function  Outflow tract obstruction  Valvular function & flow assessment |
| Cardiac tumour | Tumour characterisation  Ventricular volume & function  Ventricular scarring or fibrosis  Outflow tract and valvular function |
| Kawasaki disease | Coronary morphology (consider CT)  Myocardial scarring or fibrosis  Ventricular volume & function  Myocardial perfusion |
| Anomalous coronary arteries | Coronary morphology  Myocardial scarring or fibrosis  Ventricular volume & function  Myocardial perfusion |

(ASD: Atrial septal defect, VSD: Ventricular septal defect, PAPVD: Partial anomalous pulmonary venous drainage, PA: Pulmonary artery, CoA: Coarctation aorta, BCPC: Bidirectional cavo-pulmonary connection, TCPC: Total cavo-pulmonary connection, BT: Blalock-Taussig shunt, PA/VSD: Pulmonary atresia with ventricular septal defect, TGA: Transposition of the great arteries, HCM: Hypertrophic cardiomyopathy, DCM: Dilated cardiomyopathy, RV: Right ventricle)
