# Supplementary material for: The Role of Cardiovascular Magnetic Resonance in Pediatric Congenital Heart Disease
Source: J Cardiovasc Magn Reson. 2011 Sep 21;13(1):51. doi: 10.1186/1532-429X-13-51 (PMC3210092; doi:10.1186/1532-429X-13-51)
Supplement: Additional file 3 — Table S3. Example of the standard sequences and views of a usual pediatric congenital cardiac scan, in the order of workflow. [file 1532-429X-13-51-S3.DOC]

|  | **Sequence** | **Planning** | **1^o^ Purpose** | **2^o^ Purpose** |
| --- | --- | --- | --- | --- |
| **Scout** | Single shot balanced steady-state free precession images | - Multiple slices in all 3 radiological planes | Isocentering of the heart in the scanner. |  |
| **Axial black blood slices** | Respiratory-navigated, ECG-gated, ‘black-blood’ images (ultrafast spin echo or turbo spin echo).  Contiguous axial slices. | - Coverage from liver to neck - Include aortic arch & proximal branches - Include systemic & pulmonary veins. | Planning subsequent cine imaging planes. | Provides a map of thoracic anatomy. |
| **Ventricular long-axis (right and left)** | Breath-held, ECG-gated, balanced steady-state free precession cine images. | - From axial stack - Place perpendicular plane through long axis of ventricle, from mid-atrioventricular valve to ventricular apex. | Planning the true 4-chamber image. | Assessment of anterior & inferior myocardium, atrioventricular valves, ventricular sizes. |
| **Atrioventricular Valves** | Breath-held, ECG-gated, balanced steady-state free precession cine image. | - From axial stack - Place perpendicular plane parallel to, & on apical side of atrioventricular valves. - Check orientation is parallel to the vertical axis of the atrioventricular valves on left ventricular long axis & right ventricular long axis views. - The image should include base of aortic valve in systole. | Planning the 4-chamber and LV outflow tract images. | Subjective evaluation of atrioventricular valve function. |
| **4-chamber view** | Breath-held, ECG-gated, balanced steady-state free precession cine image. | - From atrioventricular valves view - Place perpendicular plane across both atrioventricular valve orifices. - From left ventricular long axis cine check that this plane passes through mid-mitral valve and LV apex. - From right ventricular long axis view check that the plane passes through mid-tricuspid valve and RV apex. | Subjective assessment of atrial volumes, biventricular volumes & function, ventricular wall motion, atrioventricular valve regurgitation. | Planning short axis stack. |
| **Short axis or trans-axial stack to cover ventricular volume** | Breath-held, ECG-gated, balanced steady-state free precession cine image. | - Contiguous slices are placed to cover the entire ventricular mass. - For axial coverage plan trans-axial slices from diaphragm inferiorly to outflow tracts superiorly. - For SAX coverage, plan from end-diastolic frame of 4-chamber cine. - Place image plane perpendicular to interventricular septum, and parallel to both AV valves, in both vertical long axis and 4-chamber views. - Extend slices to include the entire basal and apical ventricular blood pool in diastolic frame. | Provides the images required for segmentation of ventricular volumes. | Assessment of the ventricular septum, ventricular myocardial morphology & wall motion abnormalities, outflow tracts. |
| **MR angiogram** | Breath-held, not ECG-gated.  Gadolinium injection 0.2-0.4mL/kg.  Infants: injection rate 2mL/s with 5mL flush.  Older children: injection rate  3mL/s, 10mL flush. | - Isotropic voxels (1.1-1.6mm) - Planned on axial ultrafast spin echo stack, for coronal-orientated raw data. - Include antero-posterior chest wall, lung fields. - Image acquisition triggered with bolus-tracking to ensure maximum signal in structure of interest. - Two acquisitions routinely acquired, with no interval in young children, or a 15sec interval in older children. | Angiographic views of large and small thoracic vessels. Images less subject to artifact caused by low velocity or turbulent flow.  The second pass acquisition allows assessment of systemic and pulmonary venous anatomy. | Subjective determination of preferential blood flow.  Can be expanded to perform time-resolved angiography or 4-dimensional angiography. |
| **3D balanced steady-state free precession** | Free breathing, respiratory navigated, ECG-gated.  Data acquisition optimized to occur in diastole.  Signal improved following gadolinium injection.  Signal improved in tachycardic patients by triggering acquisition with every 2^nd^ heartbeat.  Acquisition time 8-15 mins. | - Planned on axial ultrafast spin echo stack for sagittal orientation of raw data. - Isotropic voxels (1.1-1.6mm). - Respiratory navigator placed mid-right dome of diaphragm, avoiding cardiac region of interest. | Provides high-resolution images of intracardiac anatomy, including coronary arteries.  Allows multiplanar reformatting. | Planning further imaging planes in patients with complex anatomy. |
| **LV outflow tract** | Breath-held, ECG-gated, balanced steady-state free precession cine image. | - From the atrioventricular valves cine. - Place a perpendicular plane through both basal aortic valve and mid-mitral valve orifice. - Check orientation passes through LV apex using left ventricular long axis cine. - Cross-cut this view to obtain two orthogonal cine views of LV outflow tract. | Outflow tract morphology, subjective assessment of semilunar valve function. | Planning phase contrast velocity mapping.  Planning “enface” view of semilunar valve. |
| **RV outflow tract and branch pulmonary arteries** | Breath-held, ECG-gated, balanced steady-state free precession cine image. | - From axial stack. - Place perpendicular plane through the pulmonary trunk. Cross-cut this view to obtain two orthogonal cine views of RV outflow tract. - Place perpendicular plane through the right and left pulmonary arteries respectively, or using multiplanar reformatting from 3D data. Cross-cut these views to obtain orthogonal longitudinal pulmonary artery cines | Outflow tract morphology.  Subjective assessment of semilunar valve function | Planning phase contrast velocity mapping.  Planning “enface” view of semilunar valve. |
| **Phase contrast flow mapping**  **Great artery flow** | Non-breath held, ECG-gated.  Through-plane phase contrast velocity mapping. | - From the orthogonal outflow tract images. - Place a perpendicular plane across the vessel of interest. - Place plane just distal to valve leaflets in systole, to avoid turbulent areas of flow. - Optimise velocity encoding to maximize accuracy and prevent aliasing. | Vessel flow volume.  Calculate regurgitant fractions (RF%).  Validate ventricular stroke volume measurements. | Calculate pulmonary blood flow to systemic blood flow ratio (Qp:Qs),  Evaluate presence and location of shunts.  Calculate flow velocity. |
| **Phase contrast flow mapping**  **Venous flow** | Non-breath held, ECG-gated.  Through-plane phase contrast velocity mapping | - From the orthogonal venous anatomy images. - Place a perpendicular plane across the vessel of interest. - Place plane between venous vessel confluence and atrial connection - Velocity encoding in region 60-100cm/s. | Venous flow volume  Calculate net pulmonary blood flow.  Calculate pulmonary arterio-venous collateral flow.  Calculate veno-veno collateral flow. | Portray sites of venous stenoses  Demonstrate direction of flow in venous collateral vessels |
| **Late gadolinium enhanced images and first pass myocardial perfusion are added to this protocol in cases with high suspicion of coronary compromise, or suspected disease or injury involving the coronary arteries.** | | | | |

**Table S3** – Example of the standard sequences and views of a usual pediatric congenital cardiac scan, in the order of workflow.
