# Supplementary figures and images for: The Role of Cardiovascular Magnetic Resonance in Pediatric Congenital Heart Disease
Source: J Cardiovasc Magn Reson. 2011 Sep 21;13(1):51. doi: 10.1186/1532-429X-13-51 (PMC3210092; doi:10.1186/1532-429X-13-51)

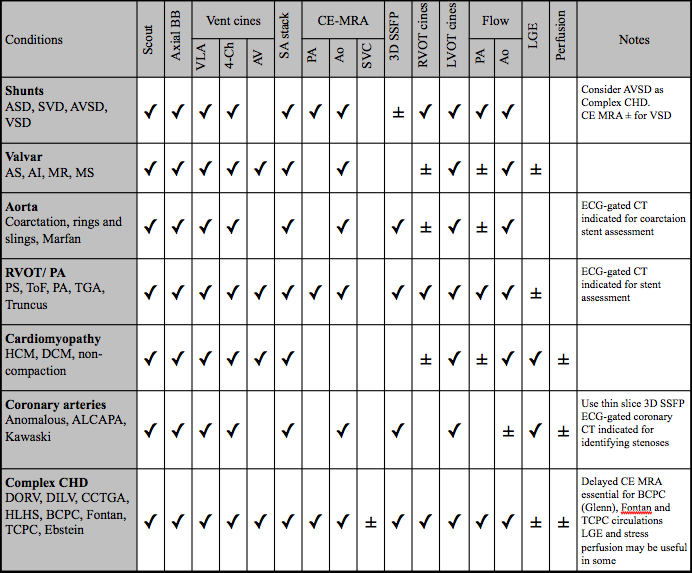


**Table S4** – Sequences that are useful in various clinical conditions.

Supplement: Additional file 4 — Table S4. Sequences that are useful in various clinical conditions. [file 1532-429X-13-51-S4.DOC]
